# Weiterentwicklung im Katastrophenschutz: Ziel, Strategie und Taktik am Beispiel der Hochwasserkatastrophe 2021 im Ahrtal: „Lessons learned“ aus Sicht der Medizinischen Task Force 47 (Unterfranken)

**DOI:** 10.1007/s10049-022-01089-7

**Published:** 2022-11-11

**Authors:** Maximilian Kippnich, Uwe Kippnich, Harald Erhard, Patrick Meybohm, Thomas Wurmb

**Affiliations:** 1 Klinik und Poliklinik für Anästhesiologie, Intensivmedizin, Notfallmedizin und Schmerztherapie, Sektion für Notfall- und Katastrophenmedizin, Universitätsklinikum Würzburg, Oberdürrbacher Str. 6, 97080 Würzburg, Deutschland; 2 Landesgeschäftsstelle, Bayerisches Rotes Kreuz, München, Deutschland; 3 Bezirksgeschäftsstelle Unterfranken, Bayerisches Rotes Kreuz, Würzburg, Deutschland; 4 Klinik und Poliklinik für Anästhesiologie, Intensivmedizin, Notfallmedizin und Schmerztherapie, Universitätsklinikum Würzburg, Würzburg, Deutschland

**Keywords:** Hochwasser, Sturzflut, Katastrophenschutz, Medizinische Task Force, Kritische Infrastruktur, Flooding, Sudden flood, Civil protection, Medical task force, Critical infrastructure

## Abstract

**Hintergrund und Fragestellung:**

Im Rahmen der überörtlichen Katastrophenhilfe war die Medizinische Task Force 47 (Unterfranken) im Juli 2021 bei der Hochwasserkatastrophe in Rheinland-Pfalz (Ahrtal) im Einsatz. Mit dem Ziel, mögliche Verbesserungen im Katastrophenschutz aus Sicht einer überregionalen Einheit aus dem Einsatz ableiten zu können, wurde dieser wissenschaftlich evaluiert und die Erkenntnisse in einen übergeordneten Kontext gesetzt.

**Material und Methoden:**

Nach Definition eines konkreten Auswerteprozesses wurden durch ein interdisziplinäres Expertengremium Einsatzunterlagen und relevante Konzepte des Bayerischen Roten Kreuzes gesichtet. Auf dieser Basis wurden Strategien und Taktiken entwickelt, um die vordefinierten Ziele zu erreichen.

**Ergebnisse:**

Die Leistungsfähigkeit der Einsatzeinheiten könnte durch moderne Einsatzmittel (E-Bike, Drohnen, hochgeländegängige Fahrzeuge) gesteigert werden. Zur Erhöhung der Reaktionsfähigkeit könnten neue Schnell-Einsatz-Gruppen (SEG) erforderlich sein, die als Teil von BOS-übergreifenden Erkundungseinheiten agieren und in unwegsamen Geländen autark Einsatzaufträge abarbeiten können (SEG Erkundung und SEG Gelände-Infrastruktur-Logistik-Transport). Die taktischen Einheiten könnten in die regionale und überregionale Katastrophenhilfe eingebunden und synchronisiert werden.

**Diskussion:**

Für den weitestgehend ehrenamtlich organisierten Katastrophenschutz in Deutschland könnte es in Zukunft erschwert sein, die Vielzahl möglicher modernster Einsatzmittel im hochkomplexen Umfeld einer Katastrophe sicher einsetzen zu können. Eine Teilprofessionalisierung durch hauptamtliche Führungs- und Einsatzkräfte könnte eine Lösung hierfür sein.

## Abstract (en)

**Objectives:**

In July 2021, the Medical Task Force 47 was deployed to the flood disaster in western Germany as part of the cross-regional disaster relief system. With the aim of deriving possible improvements in disaster management, it was scientifically evaluated. The focus of this study was the strategy to build up and to maintain a responsive and efficient rescue system.

**Materials and methods:**

After defining an evaluation process, an interdisciplinary panel of experts reviewed operational documents and relevant concepts of the Bavarian Red Cross. Based on these, strategies and tactics were developed to achieve predefined goals.

**Results:**

The performance of the emergency units could be increased by modern vehicles and equipment (eBikes, drones, all-terrain vehicles). To improve the response capability, new rapid response groups could be required that can take over the function of an advance command and can process mission assignments in impassable terrain autonomously. These could be integrated and synchronized into regional and cross-regional disaster relief.

**Conclusion:**

In future, it could be difficult for Germany’s largely volunteer-organised disaster management to safely deploy the multitude of possible state-of-the-art resources in the highly complex environment of a disaster. Partial professionalization by full-time command and control staff could be one solution for this problem.

## Einleitung

Die Hochwasserkatastrophe im Juli 2021 in Nordrhein-Westfalen und Rheinland-Pfalz war eine dynamische Flächenlage mit einer hohen Anzahl an Toten und Verletzten, einem hohen Maß an menschlichem Leid und einer nachhaltigen Schädigung der kritischen Infrastruktur. Neben einer Vielzahl von regionalen und überregionalen Einsatzkräften war u. a. die Medizinische Task Force 47 (MTF) im Einsatz. In der folgenden Arbeit werden aus der Perspektive der MTF 47 mögliche Konzepte zur Verbesserung des Katastrophenschutzes der Zukunft dargestellt. Diese Konzepte basieren auf allgemeinen Erwägungen ergänzt durch die Erfahrungen aus dem Einsatz im Ahrtal.

Im Rahmen der überregionalen Katastrophenhilfe wurde am 17.07.2021 im Auftrag des Bayerischen Staatsministeriums des Innern, für Sport und Integration die MTF 47 alarmiert. Die MTF 47 entspricht dem Hilfeleistungskontingent Standard Unterfranken (bayerische Begrifflichkeit). Eine MTF ist eine bundeseinheitliche taktische Einheit mit dem Schwerpunkt des (überregionalen) medizinischen Katastrophenschutzes. Jede MTF besteht aus 5 Teileinheiten: Führungsgruppe, Dekontaminationszug für Verletzte, Behandlungsbereitschaft, Patiententransportgruppe und Logistikzug. Deutschlandweit gibt es 61 MTF [1]. Der initiale Einsatzauftrag war die notfallmäßige Verlegung in den Bereitstellungsraum am Nürburgring. In einzelnen Gebieten im Raum Ahrweiler wurde die sanitäts- und betreuungsdienstliche Versorgung der betroffenen Bevölkerung und Einsatzkräfte anderer Fachdienste übernommen. Hierzu zählten u. a. die Einrichtung und das Betreiben von (ortsfesten) Sanitätsstationen und die Unterstützung des Regelrettungsdiensts. Ein weiterer Einsatzschwerpunkt lag auf der Errichtung von Feldküchen und der Versorgung mit Warmverpflegung. Im Rahmen des Einsatzes waren verschiedene Erkundungseinsätze erforderlich. Die MTF 47 war die erste bayerische Einheit im Katastrophengebiet und war mit 147 Einsatzkräften und 40 Fahrzeugen vom 17.07.2021 bis zum 20.07.2021 im Einsatz. Die Einsatzkräfte waren in einer Schule in der Nähe des Schadensgebiets untergebracht, die Selbstversorgung erfolgte entsprechend der Rahmenkonzeption für MTF vollständig autark [1].

Ziel der Arbeit ist es, anhand einer übergeordneten und genau definierten Analysestruktur die konkreten Einsatzerfahrungen der MTF 47 als überörtlich zugeführter Einheit systematisch darzustellen, zu bewerten und ggf. Lösungsvorschläge zur Weiterentwicklung katastrophenmedizinischer Einsatzkonzepte zu erarbeiten.

### Qualitätsmanagement, kontinuierliche Überprüfung und Weiterentwicklung des Katastrophenschutzes

Das Lernen aus Katastrophen ist essenziell, um das gesamte System des Katastrophenschutzes weiterzuentwickeln. Hierzu bedarf es eines strukturierten Prozesses, der die Auswertung von realen Einsätzen zum Ziel hat. Erforderlich hierzu wäre zuvorderst die Definition von Qualitätsindikatoren, um zum einen Einsätze vergleichbar zu machen, zum anderen aber auch, um eine standardisierte und objektivierbare Auswertung mit dem Ziel einer systematischen Weiterentwicklung durchzuführen [2, 3]. Ein solcher Prozess wurde beispielsweise erfolgreich bei der Auswertung des Würzburger Terroranschlags (Axtattentat im Regionalexpress von 2016) durchgeführt [4], außerdem wurde ein Forschungsprojekt des Instituts für Rettungs- und Notfallmedizin des Universitätsklinikums Schleswig-Holstein (QUARZ-SAND) zur Erarbeitung von Qualitätsindikatoren bei Katastropheneinsätzen erfolgreich abgeschlossen [5].

**Figure 1:**
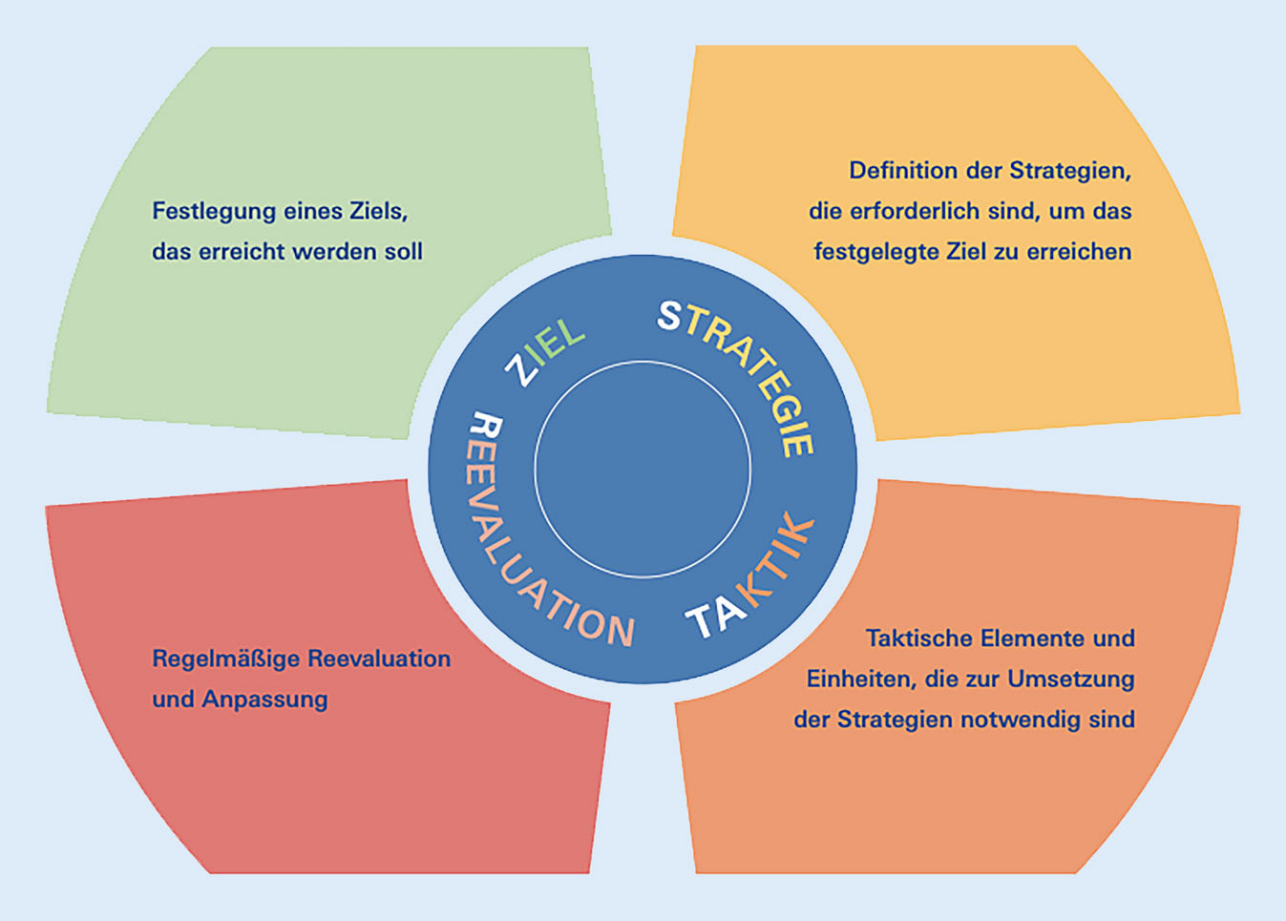

Auch das oben beschriebene Schema aus übergeordneten Zielen, den dazugehörigen Strategien und taktischen Elementen muss regelmäßig auf seine Gültigkeit hin überprüft werden. Dies kann anhand eines Zyklus erfolgen, der ähnlich dem PDCA-Zyklus immer wieder durchlaufen wird – des *ZSTAR-Kreises* (*Z*iel, *S*trategie, *Ta*ktik und *R*eevaluation; Abb. 1). Dieser wurde erstmalig 2022 im Rahmen einer Anhörung im bayerischen Landtag von Prof. Wurmb verwendet. Der ZSTAR-Kreis könnte insbesondere für Einsatzvorbereitung und Konzeptentwicklung hilfreich sein. Davon abzugrenzen ist die Dienstvorschrift 100 (Führen im Einsatz), welche zur Führung im Einsatz selbst angewandt wird [6]. Die Nutzung des bekannten PDCA-Zyklus oder anderer Regelkreise wäre prinzipiell denkbar [7]. Allerdings handelt es sich bei dem ZSTAR-Kreis um ein Werkzeug, bei dem die Zielsetzung wesentlicher Teil des Regelkreises ist und nicht außerhalb des Zyklus darübersteht. Damit wird der ganz konkrete Bezug zur Planung und Bewältigung katastrophenmedizinischer Einsätze hergestellt (s. Methodik), die Steuerung von QM-Maßnahmen tritt hierbei gänzlich in den Hintergrund.

### Fragestellung

In der vorliegenden Arbeit sollen mithilfe des „ZSTAR“-Kreises aus dem beschriebenen Einsatz der MTF 47 die bestehenden Strategien und Taktiken überprüft bzw. neu entwickelt werden.

## Methodik

Zunächst ist die Darstellung eines übergeordneten Systems notwendig, um die Gewinnung der Erkenntnisse und Ergebnisse besser einordnen zu können.

### Systematische Betrachtung zur Planung katastrophenmedizinischer Einsätze: Ziel – Strategie – Taktik – Reevaluation

*Generelle Ziele* im medizinischen Katastrophenschutz lauten:

1. Prävention von Schadensereignissen, die eine Wirkung auf Gesundheit und gesellschaftliche Integrität entfalten
2. Erhalt und schnellstmögliche Wiederherstellung von Gesundheit und gesellschaftlicher Integrität nach Eintreten eines Wirkung entfaltenden Schadensereignisses

Die generelle *Strategie* zum Erhalt und zur Wiederherstellung der Gesundheit nach Eintritt eines Schadensereignisses besteht aus zwei wesentlichen Komponenten:

1. Einwirkung reduzieren durch Vorhandensein von Warn- und Schutzsystemen
2. Auswirkungen bewältigen durch Aufbau und Vorhalten eines reaktions- und leistungsfähigen Rettungs- und Gesundheitssystems, das kontinuierlich an den aktuellen Stand der Wissenschaft und der Technik angepasst werden muss

Im folgenden Schritt wird auf die taktischen Elemente, Mittel und Werkzeuge eingegangen, die zur Umsetzung dieser Strategie prinzipiell benötigt werden. Die vorliegende Aufzählung geht über den rein sanitätsdienstlichen Aspekt hinaus, erhebt aber keinen Anspruch auf Vollständigkeit und ist als eine mögliche Auswahl zu verstehen.

*Taktik*, Mittel und Werkzeuge zur Umsetzung der Strategie:

I. Einwirkung reduzieren:

1. Etablierung von Warnsystemen, z. B.:
  Akustische Systeme, z. B. Sirenen mit einem einfachen Code, der eine klare und uniforme Reaktion und Verhaltensweise der gewarnten Bevölkerung triggert
  IT-gesteuerte Systeme, z. B. Warnnachrichten auf Mobiltelefone über Apps oder SMS, automatisch funktionierende Warn-Apps
  Traditionelle und IT-unabhängige Systeme, z. B. Radio- oder Lautsprecherdurchsagen
2. Aufbau von Schutzsystemen, z. B.:
  Siedlungs- und Gebäudestruktur
  Evakuierungspläne und Ausweisen von Sammelstellen
  Vorplanen von Schutzräumen
  Aufbau und Vorhaltung der Versorgung mit lebenswichtigen Gütern (z. B. Wasser, Nahrung, Kleidung für die Bevölkerung)
  Steigerung der Ressourcen für Themen des Katastrophenschutzes in Kreisverwaltungsbehörden

II. Auswirkungen bewältigen:

1. Aufbau und Vorhalten eines reaktions- und leistungsfähigen behörden- und organisationsübergreifenden Rettungssystems
  Vorhalten leistungsfähiger taktischer Einsatzeinheiten, z. B.:
    a) Ausgebildetes und trainiertes Personal (operativ und administrativ auf allen Leitungs- und Führungsebenen)
    b) Schutzausrüstung nach aktuellem Stand der Technik
    c) Technische Ausstattung nach aktuellem Stand der Technik
    d) Einsatztauglicher Fuhr- und Gerätepark, adaptiert an das antizipierte Ereigniserfordernis (z. B. durchhaltefähig und geländetauglich)
  Darstellung und damit Kenntnis über die vorhandenen Fähigkeiten verfügbarer Einheiten
  Festlegung und Kommunikation der Abruf- und Anforderungswege der Einheiten
  Synchronisierte Führungsebenen, v. a. bei überregionalen Einsätzen
  Darstellung der Führungsebenen unterschiedlicher Systeme in Äquivalenztabellen (sowohl föderal als auch organisationsbezogen, u. a. Militär für die zivilmilitärische Zusammenarbeit)
  Länderübergreifende gemeinsame Sprache durch die Verwendung gleicher Begrifflichkeiten oder die Erstellung von Äquivalenztabellen (Harmonisierung über die Innenministerkonferenz)
  Einheitliches System zur Lagedarstellung, z. B. Windmühlenmodell [8]
  Einführung und Nutzung einer gemeinsamen Kommunikationsplattform
2. Aufbau und Vorhaltung eines reaktions- und leistungsfähigen Gesundheitssystems
  Betreiben von leistungsfähigen Krankenhäusern, die in Planung und Übungen des Katastrophenschutzes eingebunden werden und ihre Aufgabe im Rahmen von Großschadenslagen und Katastrophen kennen und wahrnehmen müssen (Anmerkung: Die SARS-CoV-2-Pandemie hat die elementare Bedeutung der Krankenhäuser deutlich gezeigt.)
    a) Weiter als bisher reichende gesetzliche Verankerung der Krankenhaus-Alarm- und -Einsatzplanung (KAEP)
    b) Regelung der Finanzierung der KAEP
    c) Gesetzliche Verankerung einer verantwortlichen Leitung in Krankenhäusern für die Krankenhaus-Alarm- und -Einsatzplanung
    d) Etablierung einer Alarmkette für Krankenhäuser und verbindliche Festlegung einer Kontaktfunktion bei Schadenslagen
    e) Etablierung von Alarmketten zwischen den Krankenhäusern
    f) Etablierung von Patienten- und Ressourcensteuerung zwischen den Krankenhäusern (vgl. Ärztlicher Leiter Krankenhauskoordinierung Corona Bayern, Einbindung in den Katastrophenstab der Kreisverwaltungsbehörden)
    g) Einsatz von ärztlichem Personal aus Krankenhäusern in Krisengebieten (Ärztetrupps) muss im Vorfeld geplant, organisiert und finanziert werden (z. B. gesetzliche Regelung)
  Überarbeitung der Sanitätsmittelbevorratung des Bundes und der Länder

### Methodik zur Entwicklung der Ergebnisse aus den Erfahrungen der MTF 47

Es wurde in den ersten 2 Monaten nach Rückkehr der MTF 47 aus dem Einsatzgebiet ein interdisziplinäres und multiprofessionelles Expertengremium aus Mitarbeitern der Sektion für Notfall- und Katastrophenmedizin und aus Leitungs- und Führungskräften des Bayerischen Roten Kreuzes Unterfranken (BRK) gebildet. Daraufhin wurden zunächst die konkreten Inhalte des Auswerteprozesses definiert. Insbesondere die Strategie „*Auswirkungen bewältigen durch Aufbau und Vorhalten eines reaktions- und leistungsfähigen Rettungssystems*“, das kontinuierlich an den aktuellen Stand der Wissenschaft und der Technik angepasst werden muss, sollte hierbei überprüft werden. Die Evaluierung bestehender Katastrophenschutzstrukturen im Hinblick auf überregionale Einsätze war hierbei von besonderer Relevanz.

Es wurden hierzu sämtliche relevanten Konzepte des BRK und das Rahmenkonzept Medizinische Task Force des Bundesamts für Bevölkerungsschutz und Katastrophenhilfe (Tab. 1) sowie die aktuellen Einsatzunterlagen gesichtet. Zu Letzteren zählten Einsatztagebücher, Lagemeldungen und Vorträge, Einsatzprotokolle sowie Presseberichte (Tab. 2).

**Table 1:** 

Konzept	Jahr der Veröffentlichung
Auftrags- und Alarmblätter für die Alarmierung der Hilfeleistungskontingente Unterfranken	2019
Rahmenkonzept Medizinische Task Force für die Aufstellung und den Einsatz der Medizinischen Task Force	2018
Typenblatt Schnell-Einsatz-Gruppe Psychosoziale Notfallversorgung	2018
Typenblatt Schnell-Einsatz-Gruppe Behandlung	2016
Typenblatt Schnell-Einsatz-Gruppe Transport	2016
Typenblatt Schnell-Einsatz-Gruppe Betreuung	2016
Typenblatt Schnell-Einsatz-Gruppe Verpflegung	2016
Typenblatt Schnell-Einsatz-Gruppe Technik und Sicherheit	2016
Typenblatt Schnell-Einsatz-Gruppe Information und Kommunikation	2016
Typenblatt Schnell-Einsatz-Gruppe Rettungshunde	2016
Typenblatt Schnell-Einsatz-Gruppe CBRNE	2016
Checkliste Kontingentansprechpartner Kreisverband bei Kontingentalarm Unterfranken	2013
Taktisches Schaubild Hilfeleistungskontingent Unterfranken	2013
Karte Standorte Kontingente in Bayern	2011
Funkrufnamen der Hilfeleistungskontingente	2010
Sammelräume für Hilfeleistungskontingente Unterfranken	2010
Planungsrichtlinien für die Aufstellung von Hilfeleistungskontingenten im Sanitäts- und Betreuungsdienst zur überregionalen bzw. länder- und staatenübergreifenden Katastrophenhilfe	2009
Richtlinie für den Sanitäts- und Betreuungsdienst des Katastrophenschutzes der Hilfsorganisationen in Bayern	2009
Richtlinie für die Alarmierung Sanitäts- und Betreuungsdienst des Katastrophenschutzes der Hilfsorganisationen in Bayern	2009
Kurzübersicht Wasserrettungszug Unterfranken	2007
Taktisches Schaubild Ablauf bei Einsätzen im Rahmen des EU-Gemeinschaftsverfahrens	2006
Taktisches Schaubild länderübergreifende Katastrophenhilfe	2004
Alarmierungsstufen bei Großschadensfällen oder im K‑Fall	2002

**Table 2:** 

Art	Anzahl	Beschreibung
Einsatztagebuch	3	Rückwärtiger Führungsstab
		Kontingentführung
		Schnell-Einsatz-Gruppe Information und Kommunikation des Kontingents
Lagevortrag	10	Führungsstab Kontingent
Pressebericht	12	Printmedien
		Online-Berichte
		Radiointerviews
		TV-Berichterstattung

Basierend auf diesen Unterlagen und Erfahrungsberichten wurden vom Expertengremium Strategien und Taktiken entwickelt, mit denen die o. g. Ziele erreicht werden könnten. Die Ergebnisse wurden von allen beteiligten Experten konsentiert und wurden in innerverbandliche Gremienarbeit (BRK) sowie auf regionalen und überregionalen Veranstaltungen eingebracht. So sind die Ergebnisse beispielsweise in einer Expertenanhörung im Innenausschuss des Bayerischen Landtags vorgestellt worden.

Die aus diesem Prozess gewonnenen Erkenntnisse und Lektionen werden im Folgenden unter den Ergebnissen in das oben definierte Schema Ziel-Strategie-Taktik eingeordnet. Der Fokus hierbei liegt auf der Strategie der Bewältigung durch den Aufbau und das Vorhalten eines reaktions- und leistungsfähigen behörden- und organisationsübergreifenden Rettungssystems.

## Ergebnisse

### Ziel.

Erhalt und schnellstmögliche Wiederherstellung von Gesundheit und gesellschaftlicher Integrität nach Eintreten eines Wirkung entfaltenden Schadensereignisses.

### Strategie.

Auswirkungen bewältigen durch Aufbau und Vorhalten eines reaktions- und leistungsfähigen behörden- und organisationsübergreifenden Rettungssystems.

### Taktisches Mittel 1.

Vorhalten leistungsfähiger taktischer Einsatzeinheiten

- Trainiertes Personal
- Schutzausrüstung nach aktuellem Stand der Technik
- Technische Ausstattung nach aktuellem Stand der Technik
- Einsatztauglicher Fuhr- und Gerätepark, adaptiert an das antizipierte Ereigniserfordernis (z. B. durchhaltefähig und geländetauglich); BOS-übergreifende Abstimmung zur gemeinsamen Nutzung auch unter Einbeziehung militärischer Aufklärungsmittel (z. B. Luftfahrzeugunterstützung durch Hubschrauber der Polizei und der Bundeswehr)

### Bewertung.

Die bestehenden Strukturen im Katastrophenschutz wie z. B. die Schnell-Einsatz-Gruppe Behandlung und die Schnell-Einsatz-Gruppe Transport haben sich bei der notfallmedizinischen Versorgung von Verletzten bewährt. Im Ahrtal 2021 waren für die MTF 47 die Lageerkundung, das unwegsame Gelände sowie die Flexibilität der Einheiten die führende Herausforderung.

### „Lesson identified“ 1.

Zur Steigerung der Einsatzfähigkeit in unwegsamem Gelände könnten moderne Einsatzmittel wie Drohne, E‑Bike, hochgeländegängiges Fahrzeug, SUV und Unimog in die bestehenden Einsatzeinheiten eingebunden werden. Alternativ könnten Fachberater aus dem Sanitäts- und Betreuungsdienst als Teil einer multiprofessionellen und interdisziplinären Erkundungseinheit agieren. Die Evaluierung dieser Einsatzmittel ist in Tab. 3 dargestellt.

**Table 3:** 

Einsatzmittel	Abbildung	Einsatzmöglichkeit
Drohne	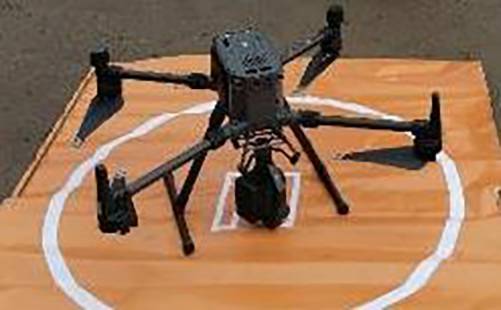	Erkundung nicht zugänglicher Gebiete Übermittlung aktueller Lagebilder Transport (kleiner Hilfsgüter), z. B. Verbandmittel, Rettungsfolie
E‑Bike^a^	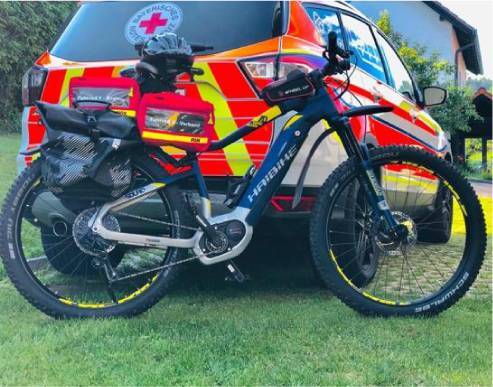	Erkundung von für Fahrzeugen nicht zugänglichen Gebieten Erstversorgung von Verletzten und zu Betreuenden
Hochgeländegängiges Fahrzeug (z. B. ARGO, Fa. Ontario Drive & Gear, New Hamburg, Kanada)	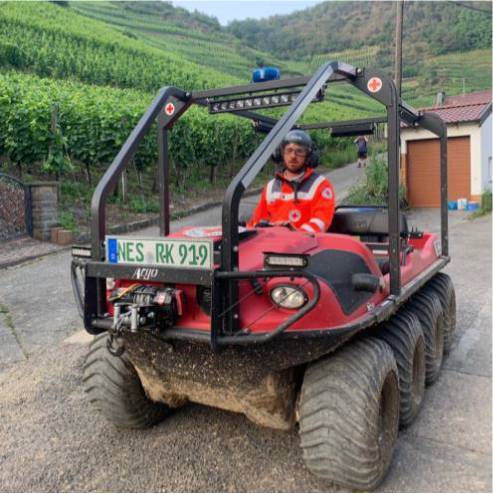	Erkundung von schwer zugänglichen Gebieten Transport von Helfern und Material (Logistik) Erweiterte Versorgung von Verletzten und zu Betreuenden Transport von Verletzten
SUV	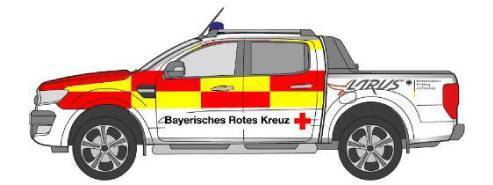	Einsatzführung Kommunikation Erkundung Transport von Helfern und Material (Logistik) Startstation für Drohnen
Unimog	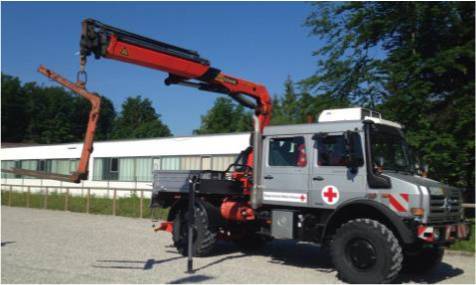	Erkundung Kommunikation Transport von Helfern und Material (Logistik) Rettung und Transport von Verletzten

^a^Der Akku müsste im Katastropheneinsatz mittels Generator geladen werden. Ein alternatives Einsatzmittel mit vergleichbarem Einsatzwert wäre ein kleines Motorrad mit Verbrennungsmotor. Hier könnte der Schulungs- und Wartungsaufwand aber höher sein

### „Lessons identified“ 2.

**Figure 2:**
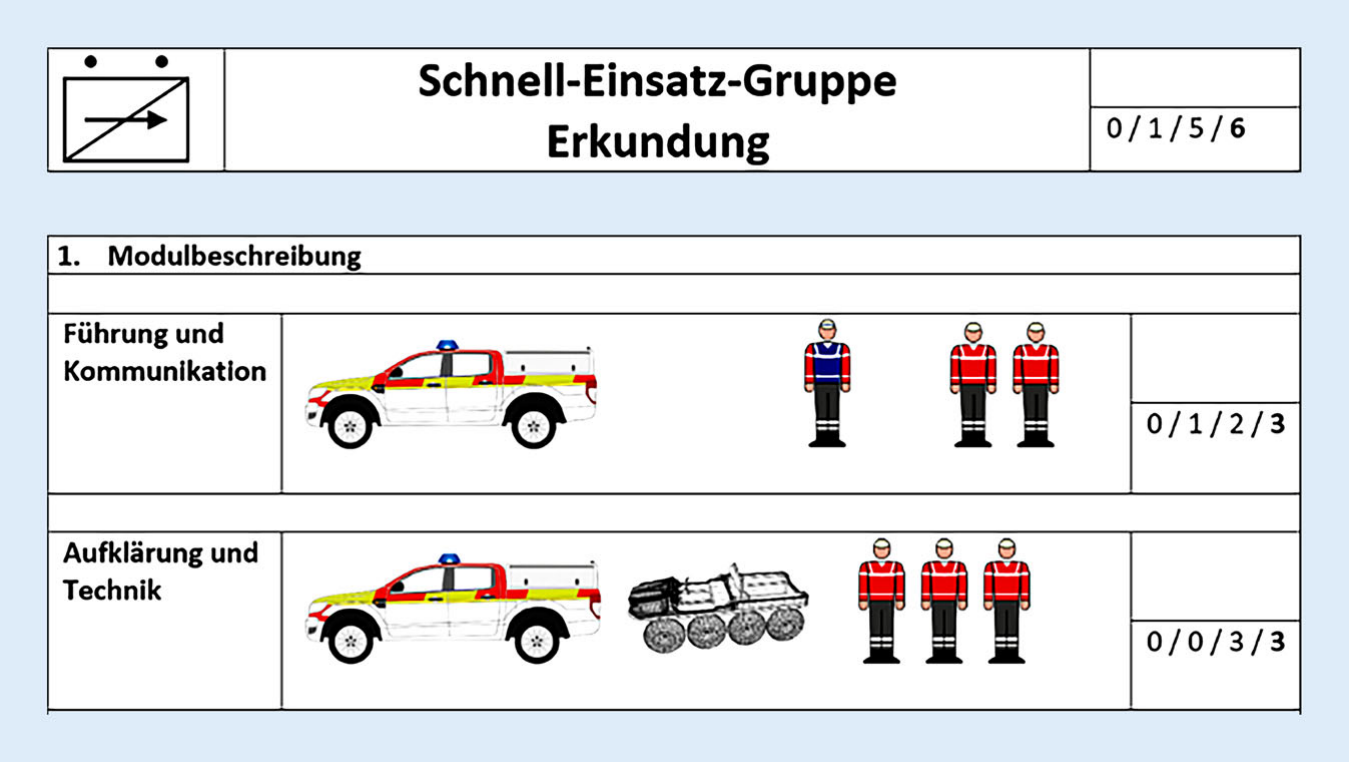

**Figure 3:**
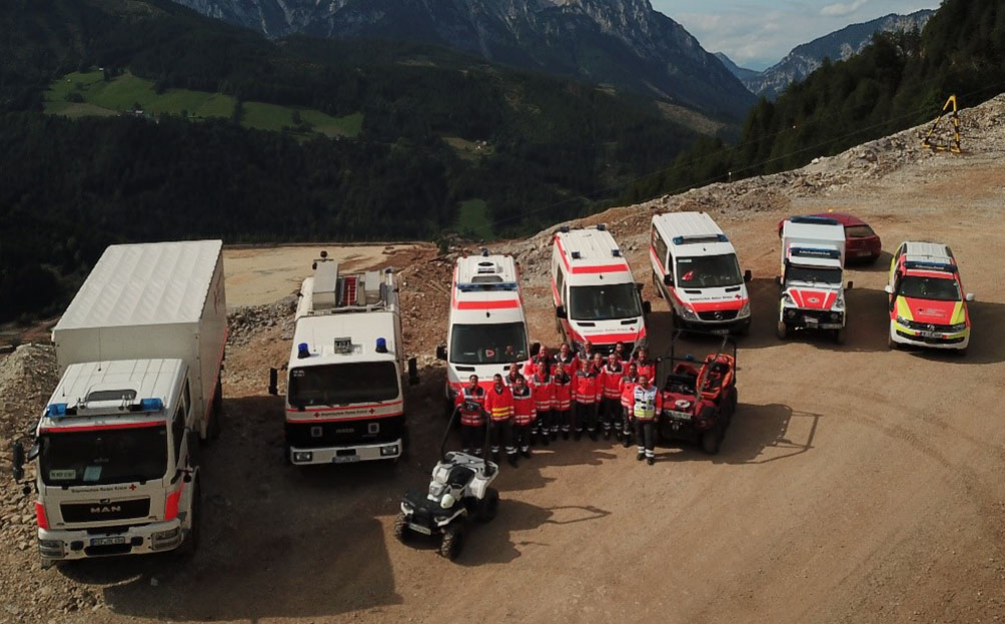

Um zukünftige Einsätze schneller und sicherer abarbeiten zu können, könnten folgende Einsatzeinheiten unter Einbezug der evaluierten Einsatzmittel hilfreich sein: Schnell-Einsatz-Gruppe Erkundung (Tab. 4; Abb. 2) und Schnell-Einsatz-Gruppe G.I.L.T. (G.I.L.T. = Gelände, Infrastruktur, Logistik, Transport; Tab. 5; Abb. 3; diese Piloteinheiten sind seit 2018 im Bayerischen Roten Kreuz Unterfranken in der Konzeption und in der ständigen Weiterentwicklung).

**Table 4:** 

Fahrzeuge	Helfer	Einsatzwert
2 SUV 1 hochgeländegängiges Fahrzeug 1 Drohne	1 Gruppenführer 5 Helfer	Schnelle Einsatzfähigkeit
		Erkundung in unwegsamem Gelände
		Führung und Kommunikation
		Logistik
		Notfallmedizinische Erstversorgung
		Menschenrettung

*SUV* sport utility vehicle (Geländewagen)

**Table 5:** 

Fahrzeuge	Helfer	Einsatzwert
2 SUV 1 Mannschaftstransportwagen 2 geländegängige KTW‑B 1 Gerätewagen Technik und Sicherheit 1 geländegängiger LKW	1 Zugführer 2 Gruppenführer 10 Helfer	Autarke Einsatzabwicklung in unwegsamem Gelände und bei zerstörter Infrastruktur mit den Schwerpunkten Gelände, Infrastruktur, Logistik und Transport

^a^Je nach verfügbaren Ressourcen und Einsatzauftrag können Fahrzeuge und Helfer angepasst werden. Die beschriebenen Einheiten stellen eine Mindestanforderung dar

### „Lesson identified“ 3.

In möglichen zukünftigen Einsatzszenarien bei Großwetterlagen, in unwegsamen Geländen und bei zerstörter Infrastruktur ist die Gefährdung für Personal und Material, aber auch Verletzte und Betroffene ein zentrales Thema. Eine Sensibilisierung muss in der Grundausbildung beginnend hierfür stattfinden. Außerdem sind praktische Übungen für das Verhalten in solchen Einsätzen erforderlich. Beispielsweise sind hier Fahrübungen im Gelände sowie notfallmedizinische Erstversorgung und Rettung unter erschwerten Bedingungen zu nennen.

### Taktisches Mittel 2.

Einbindung und Synchronisierung der taktischen Einheiten in die regionale und überregionale Katastrophenhilfe

- Darstellung und damit Kenntnis über die vorhandenen Fähigkeiten verfügbarer Einheiten
- Festlegung und Kommunikation der Abruf- und Anforderungswege der Einheiten
- Synchronisierte Führungsebenen, v. a. bei überregionalen Einsätzen
- Darstellung der Führungsebenen unterschiedlicher Systeme in Äquivalenztabellen
- Länderübergreifende gemeinsame Sprache durch die Verwendung gleicher Begrifflichkeiten oder die Erstellung von Äquivalenztabellen

### Bewertung.

Bei der Eingliederung von bayerischen Einheiten in ein außerbayerisches Führungssystem sind die außerbayerischen und bayerischen Führungsebenen weder in Funktion noch in Nomenklatur identisch. So entspricht der bayerischen Führungsgruppe Katastrophenschutz (FüGK) in etwa die Aufsichts- und Dienstleistungsdirektion (ADD) in Rheinland-Pfalz. Der Sanitätseinsatzleitung (SanEL), wie sie in Bayern definiert ist, entspricht in Rheinland-Pfalz in etwa die Abschnittsleitung Gesundheit (ALG), die zudem noch eine nicht vergleichbare Position und Stellung im Führungssystem hat. Diese Unterschiede erschweren die Einhaltung der Melde- und Kommunikationswege und führen zu Zeitverlust und verzögerter Einbringung der Hilfeleistung in den konkreten Einsatz.

### „Lesson identified“ 4.

Die Medizinischen Task Forces in Deutschland sind gut etabliert und haben sich bewährt. Aufgrund der Vielzahl der Helfer und Fahrzeuge und der Ehrenamtlichkeit haben MTF eine gewisse Vorlaufzeit. Bei Unwetterlagen wie Sturzfluten ist ein schnelles, effizientes und überregionales Eingreifen essenziell. Die im Folgenden aufgeführte Taktik soll die MTF ergänzen und ihnen vorgeschaltet sein. Diese Taktik kann aber auch bei lokoregionalen Lagen in modifizierter Form angewandt werden.

1. Frühzeitiger Voralarm überregionaler Einheiten (auch wenn noch kein konkreter Einsatzauftrag vorliegt)
2. Baldmöglichste Entsendung eines Vorauskommandos ins Schadensgebiet, das die Hilfsorganisation, die Einsatzeinheit oder z. B. das Bundesland operativ im Schadensgebiet vertritt.
  Das Vorauskommando
  - besteht aus einer organisatorischen Führungskraft und einem leitenden Notarzt,
  - wird von Helfern einer Informations- und Kommunikationseinheit unterstützt,
  - verfügt über zwei Kommandowagen, ein hochgeländegängiges Fahrzeug und eine Drohne (z. B. SEG Erkundung),
  - hat den Auftrag, konkrete Einsatzaufträge von der örtlichen Einsatzleitung entgegenzunehmen, die dafür erforderlichen Erkundungen durchzuführen und erste Strukturen zur weiteren Hilfe zu etablieren.
3. Zeitnahes Nachführen eines geländegängigen Einsatzzugs (z. B. SEG G.I.L.T.)
  - Bildet mit der Gesamteinsatzleitung einen Verband
  - Beginnt unmittelbar mit der Einsatzabwicklung
    i. Schutzziele: Eigenschutz und Menschenrettung
  - Etabliert Strukturen für den Einsatz der nachfolgenden MTF
4. Eigentlicher Einsatz der MTF, der durch die vorgeschalteten Einheiten vorbereitet wurde
5. Vorauskommando hält engen Kontakt zur örtlichen Einsatzleitung und zur rückwärtigen Einsatzführung im entsendenden Gebiet („*single point of contact*“)
6. Im Idealfall würde man sich auf eine einheitliche länderübergreifende Nomenklatur der Führungsebenen einigen. Dies dürfte aufgrund langjährig etablierter landesspezifischer Nomenklatur schwierig sein. Alternativ könnten Äquivalenztabellen erstellt werden, die eine horizontale Vergleichbarkeit der Führungsebenen ermöglichen würden. Bei überregionalen, aber vor allem bei länder- oder landesübergreifenden Einsätzen wäre die Festlegung von Meldepunkten im Sinne eines „single point of contact“ (SPOC) für jede Führungsebene und die Gesamteinsatzführung wichtig. Die Nomenklatur dieser Meldepunkte muss eindeutig für die verschiedenen Führungsebenen gewählt und am besten zweisprachig (deutsch/englisch) ausformuliert werden.
7. Gerade im Hinblick auf überörtliche Einsätze existiert keine einheitliche, von allen Einsatzkräften nutzbare Plattform zur Kommunikation und zur Lagedarstellung. Dies erschwert eine fortgeschriebene Lagedarstellung und Bewertung aller Einsatzabschnitte. Die Anforderungen an ein solches System sind u. a. die leichte und intuitive Bedienbarkeit, die niederschwellige Zugänglichkeit und eine hohe Verfügbarkeit. Ein solches System sollte in Zukunft länderübergreifend verfügbar sein.

## Diskussion

Der Katastrophenschutz in Deutschland steht aufgrund neuer Einsatzlagen vor großen Herausforderungen und Veränderungen. Neben Extremwetterlagen und COVID-19-Pandemie haben u. a. auch Terroranschläge in Europa zu einem Umdenken geführt [2, 9–11]. Die aktuelle Sicherheitslage und der Krieg in Europa werden ebenfalls neue Herausforderungen mit sich bringen.

Der Fokus der vorliegenden Arbeit liegt auf der Erarbeitung von möglichen Verbesserungen im Katastrophenschutz der Zukunft im Hinblick auf Umweltkatastrophen. Dieser Weiterentwicklungsprozess ist Teil des kontinuierlichen Qualitätsmanagements der MTF 47 und des Bayerischen Roten Kreuzes in Unterfranken. Die subjektiven Erfahrungen der MTF 47 aus der Hochwasserkatastrophe im Ahrtal 2021 sind hierbei mit eingeflossen. Dort war im Rahmen der überörtlichen Katastrophenhilfe die MTF 47 im Einsatz. Anhand der hieraus gewonnenen Erfahrungen wurden anhand des Z‑STAR-Kreises mögliche Weiterentwicklungen im Katastrophenschutz analysiert und evaluiert. Grundgedankengeber für den Z‑STAR-Kreis war der aus dem Qualitätsmanagement bekannte Deming-Zyklus („plan-do-check-act“) sowie die Dienstvorschrift 100 (Führen im Einsatz; [6, 7]). Die Strategie „*Auswirkungen bewältigen durch Aufbau und Vorhalten eines reaktions- und leistungsfähigen Rettungssystems*“ stand hierbei im Vordergrund. Insbesondere bei den taktischen Mitteln „*Vorhalten leistungsfähiger taktischer Einsatzeinheiten*“ und „*Einbindung und Synchronisierung der taktischen Einheiten in die regionale und überregionale Katastrophenhilfe*“ konnten weitreichende Erkenntnisse gewonnen werden, welche in Teilen bereits erfolgreich in die Praxis umgesetzt werden konnten. Der Einsatz von hochgeländegängigen Fahrzeugen mit speziell trainierten Einsatzkräften nach Fernerkundung mittels Drohne ist ein Beispiel. Die erarbeiteten Konzepte können und sollen in Übungen evaluiert und entsprechend weiterentwickelt werden. Eine Folgestudie, welche die Evaluation moderner Einsatzmittel im Fokus hat, ist nach abgeschlossener Planung aktuell in der Umsetzung.

Die Bedeutung von geländegängigen Einsatzfahrzeugen und Einsatzeinheiten wird durch die Arbeit von Koks et al. hervorgehoben, in welcher die weitreichende Zerstörung der kritischen Infrastruktur durch das Hochwasserereignis 2021 in Westeuropa dargestellt wird [12]. Eine weitere Besonderheit bei der Flutkatastrophe im Ahrtal 2021 war die kurze Vorlaufzeit vor Eintritt des Ereignisses. Die hohe Zahl von mehr als 180 Toten führt Merz et al. hierauf zurück [13]. Bei solchen gravierenden und überraschenden Hochwasserereignissen sind somit schon in der Initialphase überregionale Einsatzkräfte erforderlich. Um schnell, sicher und effektiv überregionale Hilfe leisten zu können, wurde in der vorliegenden Arbeit die Etablierung von Vorauskommandos als mögliches taktisches Mittel identifiziert. Dennoch kann sich bei derartigen Katastrophenereignissen trotz Vorhandensein einer Vielzahl überregionaler Hilfskräfte die Gesamteinsatzkoordination komplex darstellen [14].

Vergleicht man die gewonnenen Erkenntnisse mit den wissenschaftlichen Arbeiten zum Hurrikan Katrina, welcher 2015 u. a. im Großraum New Orleans wütete und eine der größten Naturkatastrophen der Geschichte der Vereinigten Staaten von Amerika darstellt, lassen sich aus katastrophenmedizinischer Sicht einige Parallelen ziehen. Eine ganzheitliche Betrachtung dieser Katastrophe würde den Rahmen dieser Arbeit sprengen, weshalb der Fokus der Diskussion auf dem Management der initialen Katastrophenhilfe durch überregionale Katastrophenschutzeinheiten liegt.

Einen wichtigen Aspekt während des Hurrikans, welcher z. T. als Outcome-relevant beschrieben wird, stellte die Kommunikation zwischen den beteiligten Einheiten, Institutionen und Führungsebenen dar [15, 16]. Dies war u. a. entscheidend zur adäquaten Verteilung der benötigten Ressourcen bei der Unterbringung evakuierter Menschen. Um eine sichere Kommunikation möglichst schon in der Initialphase zu gewährleisten, könnte das beschriebene Vorauskommando mit Single-point-of-contact-Struktur die Kommunikation wesentlich vereinfachen.

Für die Einsatzabwicklung unmittelbar nach Eintritt der Katastrophe werden „disaster medical assistance teams“ und „search and rescue teams“ beschrieben, die in der Initialphase autark unter widrigen Bedingungen medizinische Nothilfe leisten können. Der Fokus dieser Einheiten ist mit dem der in der vorliegenden Arbeit beschriebenen Piloteinheiten „SEG Erkundung“ und „SEG G.I.L.T.“ vergleichbar [17].

Ein solches Vorauskommando könnte auch den Bedarf an Logistik, der für derartige Einsätze erforderlich ist, erkunden und eine frühzeitige Lageeinschätzung abgeben. Der Aufbau einer leistungsfähigen Logistik war beim Hurrikan Katrina ein weiteres zentrales Thema [18]. So war die Verteilung medizinischer Hilfsgüter von Logistikzentren aus ein wichtiges taktisches Mittel.

Erkundung und Logistik sind zentrale Punkte, welche für alle BOS gleichermaßen von Bedeutung sind und für die Abwicklung des Gesamteinsatz entscheidend sein können. Um Personal möglichst effizient einsetzen zu können und die Kernkompetenzen der verschiedenen Einheiten optimal einfließen lassen zu können, könnte eine BOS-übergreifende Erkundungsgruppe sowie Logistikeinheit das beste Vorgehen sein. Für eine zeitnahe Erkundung beispielsweise könnten polizeiliche oder militärische Aufklärungsmittel (u. a. Hubschrauber) unter Einbezug eines Fachberaters für Sanitäts- und Betreuungsdienst eingesetzt werden.

Auch wenn die Hurrikankatastrophe in 2015 und die Flutkatastrophe im Ahrtal natürlich Unterschiede aufweisen, lassen sich dennoch bei den „lessons learned“ Parallelen ziehen. Das Entscheidende bei der Betrachtung von „lessons learned“ aus vergangenen Schadenslagen ist, dass das Gelernte auch in die Praxis umgesetzt und in zukünftige Einsatzkonzepte integriert wird. Dass dies häufig nicht der Fall ist, zeigt sich beispielsweise am Einsatzmanagement von Terrorlagen: Es handelt sich bei den festgestellten Defiziten meist mehr um „lessons identified“ als um „lessons learned“ [2]. Ein Problemfeld der Weiterentwicklungen im Katastrophenschutz könnte also die unzureichende Umsetzung von „lessons identified“ aus vergangenen Großschadensereignissen und Übungen sein. Ein mögliches Hindernis für eine konkrete Umsetzung könnte u. a. der extreme Zuwachs an technischen Möglichkeiten sein. Um modernste Einsatzmittel wie beispielsweise Drohnen oder hochgeländegängige Fahrzeuge in die Gesamteinsatzstruktur einzubinden und diese sicher und effektiv zu verwenden, ist eine weitreichende Sach- und Fachkompetenz erforderlich. Im hochkomplexen Umfeld einer Katastrophe ist eine ebenso hochprofessionelle Einsatzbewältigung essenziell. Demgegenüber steht ein weitestgehend ehrenamtlich organisierter Katastrophenschutz in Deutschland. Auf ehrenamtlicher Basis wird es in Zukunft kaum möglich sein, die Vielzahl technischer Komponenten in Stand zu halten, zu beüben und im Einsatzfall zu beherrschen. Die Schere zwischen Einsatzerfordernis auf der einen Seite und demografischem Wandel mit einhergehendem Mangel an ehrenamtlichen Einsatzkräften auf der anderen Seite wird möglicherweise in Zukunft immer weiter auseinandergehen.

Eine mögliche Lösung könnte eine Teilprofessionalisierung des Katastrophenschutzes durch Etablierung hauptamtlicher Führungs- und Einsatzkräfte schon auf der Ebene der Kreisverwaltungsbehörden sein. Des Weiteren aber sollte die Stärkung des Ehrenamts im Fokus stehen. Untersuchungen zur Zufriedenheit der eingesetzten Helfer im Ahrtal 2021 zeigen, dass hier eine enorme Motivation vorhanden ist. Allerdings sind z. B. psychische Belastung und mangelnde Kommunikation identifizierte Problemfelder [19]. Um diesen Herausforderungen in Zukunft gerecht zu werden, ist ein umfassendes finanziertes Maßnahmenbündel erforderlich, welches die Stärkung des Ehrenamts zum Ziel hat.

Die Limitationen der vorliegenden Studie müssen bei der Bewertung der Ergebnisse mit einbezogen werden. Es muss hierbei bedacht werden, dass die MTF 47 nur einen sehr kurzen Zeitraum in vordefinierten Einsatzabschnitten im Gesamtgefüge der überregionalen Katastrophenhilfe im Einsatz war. Die Erfahrungen und Erkenntnisse sind vor diesem Hintergrund dementsprechend als subjektiv zu bewerten und erheben keineswegs den Anspruch auf Vollständigkeit.

## Fazit für die Praxis

Jede Katastrophenlage zeigt die gesamte Komplexität der aktuellen Herausforderungen für den Katastrophenschutz in Deutschland. Durch eine strukturierte Auswertung konnten wichtige Lektionen identifiziert werden, die zukünftig in die Taktiken und Strategien des Katastrophenschutzes integriert werden müssen, damit letztlich aus den „lessons identified“ auch „lessons applied“ werden.

## References

[1] https://www.bbk.bund.de/DE/Themen/Gesundheitlicher-Bevoelkerungsschutz/Sanitaetsdienst/MTF/mtf_node.html. Zugegriffen: 22. Sept. 2022

[2] Schorscher N, Kippnich M, Meybohm P (2022). Lessons learned from terror attacks: thematic priorities and development since 2001—results from a systematic review. Eur J Trauma Emerg Surg.

[3] Wurmb T, Franke A, Schorscher N (2020). Emergency response to terrorist attacks: results of the federal-conducted evaluation process in Germany. Eur J Trauma Emerg Surg.

[4] Wurmb T, Schorscher N, Justice P (2018). Structured analysis, evaluation and report of the emergency response to a terrorist attack in Wuerzburg, Germany using a new template of standardised quality indicators. Scand J Trauma Resusc Emerg Med.

[5] https://www.uksh.de/notfallmedizin/Das+Institut/Unser+Team/Projekte.html. Zugegriffen: 4. Mai 2022

[6] https://www.gesetze-bayern.de/Content/Document/BayVwV96946/true. Zugegriffen: 22. Sept. 2022

[7] Deming WE (1982). Out of the crisis.

[8] Wurmb T, Ertl G, Ernestus RI (2020). Command and control in hospitals during SARS-CoV-2 pandemic: the windmill model of disaster response. J Emerg Manag.

[9] Platt S, Mahdavian F, Carpenter O (2020). Were the floods in the UK 2007 and Germany 2013 game-changers?. Philos Trans A Math Phys Eng Sci.

[10] Berger E, Winkelmann J, Eckhardt H (2021). A country-level analysis comparing hospital capacity and utilisation during the first COVID-19 wave across Europe. Health Policy.

[11] Nacoti M, Ciocca A, Giupponi A (2020). At the epicenter of the Covid-19 pandemic and humanitarian crises in Italy: changing perspectives on preparation and mitigation. Catalyst.

[12] Koks E, Van Ginkel K, Van Marle M (2021). Brief Communication: critical Infrastructure impacts of the 2021 mid-July western European flood event. Nat Hazards Earth Syst Sci Discuss.

[13] Merz B, Kreiblich H, Thieken A (2021). Überraschende Hochwasserereignisse: Lehren für Risikoanalysen. Z Bevölkerungsschutz Katastrophenhilfe.

[14] Fekete A, Sandholz S (2021). Here comes the flood, but not failure? Lessons to learn after the heavy rain and pluvial floods in Germany 2021. Water.

[15] Curtis CA (2015). Understanding communication and coordination among government and service organisations after a disaster. Disasters.

[16] McSwain NE (2010). Disaster response. Natural disaster: Katrina. Surg Today.

[17] Campos-Outcald D (2006). Disaster medical response: maximizing your effectiveness. J Fam Pract.

[18] Cranmer HH (2005). Volunteer work—logistics first. N Engl J Med.

[19] https://riskncrisis.wordpress.com/publications. Zugegriffen: 6. Mai 2022

